# On the issue of psychopathological analysis of a work of fiction using the example of Dostoevsky’s “The Double”

**DOI:** 10.1192/j.eurpsy.2025.1850

**Published:** 2025-08-26

**Authors:** S. V. Motov, V. V. Motov

**Affiliations:** 1 Department of Slavic Languages & Literatures, University of Illinois Urbana-Champaign , Urbana, IL, United States; 2 Psychiatry, Social House “Stupino” (The Institution for Chronic Mental Patients with Disability) of The Department of Labor and Social Protection of the Population of the City of Moscow, Stupino, Russian Federation

## Abstract

**Introduction:**

Vladimir Chizh, who replaced Emil Kraepelin in the Department of Psychiatry at the University of Dorpat in 1891, counted no fewer than 30 characters with psychoneurological disorders in Dostoevsky’s works, beginning with the main character of “The Double,” written in 1846.

**Objectives:**

Try to answer the following questions: (1) Should a psychopathological analysis of a literary work include elements of the author’s psychobiography, psychopathological components of the author’s language, and can such work be carried out by one psychiatrist without the participation of a literary scholar? (2) What goals should such an analysis pursue? (3) Should the accuracy of the author’s description of the mental disorders present in the characters of his work be considered as a criterion for assessing the author’s artistic skill and the significance of the work as a literary and cultural phenomenon?

**Methods:**

Taking Dostoevsky’s “The Double” as a starting point, the authors analyzed professional literature on the topic and conducted their own psychopathological and literary analysis of this literary work.

**Results:**

(1) psychopathological analysis without the participation of a literary scholar is always incomplete, since everything we learn about the hero of the work we learn through the language of the work. (2) the assessment of the quality of a work of art by a psychiatrist from the point of view of the accuracy of the description of psychopathological symptoms in a particular character leads to the fact that the ideal work of art becomes a well-written case history.

**Conclusions:**

It is hardly correct to give a precise psychiatric categorization to persons whose behavior in a work of art is depicted as pathological. The author’s depiction of his hero’s pathological experiences has goals other than psychiatric ones and is conditioned by the general concept of the work.

**Disclosure of Interest:**

None Declared

